# Crystal structure of 1-ferrocenyl-2-(4-nitro­phen­yl)ethyne

**DOI:** 10.1107/S2056989020010336

**Published:** 2020-07-31

**Authors:** Sara M. Delgado Rivera, Jean C. González Espiet, Jesús M. Dones, Sebastián A. Henríquez López, Ana R. Guadalupe, Dalice M. Piñero Cruz, Ingrid Montes González

**Affiliations:** a Department of Chemistry, University of Puerto Rico at Río Piedras, PO Box 23346, San Juan, PR 00931-3346, Puerto Rico; b Department of Chemistry and the Molecular Sciences Research Center, University of Puerto Rico-Rio Piedras Campus, PO Box 23346, San Juan, 00931-3346, Puerto Rico

**Keywords:** ferrocene, 4-nitro­phenyl­ethyne, Sonogashira coupling, green chemistry, crystal structure

## Abstract

The title ferrocene derivative, [Fe(C_5_H_5_)_2_(C_8_NO_2_)], including an alkyne bonded to a *para*-nitro­phenyl substituent, which was synthesized from a copper-free Sonogashira cross-coupling reaction between ethynylferrocene and 4-bromo-1-nitro­benzene, crystallizes in the *P*2_1_/*n* space group. In the ferrocene unit, the penta­dienyl (Cps) rings are in an eclipsed conformation. The angle of rotation between the substituted cyclo­penta­dienyl ring and the *p*-nitro­phenyl group is 6.19 (10)°, yielding a quasi-linear extension of the ferrocenyl substitution. Important inter­molecular inter­actions arise from π–π stacking between the Cp rings and the *p*-nitro­phenyl, from corners of the Cp rings that are perpendicularly aligned, and between the O atoms from the nitro substituent and carbons at the corners of the Cp rings, propagating along all three crystallographic axes.

## Abstract 2

The title ferrocene derivative including an alkyne and a *para*-nitro­phenyl substitution crystallizes in the *P*2_1_/*n* space group. In the ferrocene unit, the penta­dienyl (Cps) rings are in an eclipsed conformation. Strong inter­molecular π–π-stacking, CH(Cp)—C(Cp), and O(p-nitro­phen­yl)—HC(Cp) inter­actions consolidate the crystal structure.

## Chemical context   

Recent efforts in the field of medicinal organometallic chemistry have been driven by a high inter­est in the synthesis of metal ethynyl complexes, particularly because of their biological activity (Görmen *et al.*, 2012 ▸). In addition, phenyl­ethyne-derived compounds display active electrochemical properties such as the generation of stable redox forms, regeneration at low potentials and good electrochemical reversibility (Gasser & Metzler-Nolte, 2012 ▸). 1-Ferrocenyl-2-(4-nitro­phen­yl)ethyne has previously been prepared in moderate-to-high yields (52–92%) by applying Sonogashira coupling reactions. However, all of them used 4-iodo-1-nitro­benzene or 4-triflate-1-nitro­benzene and a variety of solvents, catalysts and conditions, under an inert atmosphere. The reaction time varied from 25 min to 4 h (Torres *et al.*, 2002 ▸; Shoji *et al.*, 2014 ▸; Li *et al.*, 2009 ▸; Fu *et al.*, 2008 ▸; Coutouli-Argyropoulou *et al.*, 2003 ▸). Other approaches involved the use of iodo­ferrocene and 4-ethynyl-1-nitro­benzene (Kulhánek *et al.*, 2013 ▸). Our approach focuses on performing copper-free Sonogashira coupling between ethynylferrocene and 4-bromo-1-nitro­benzene without the need of inert atmosphere protocols and obtaining moderate-to-high yields, by following green chemistry protocols.
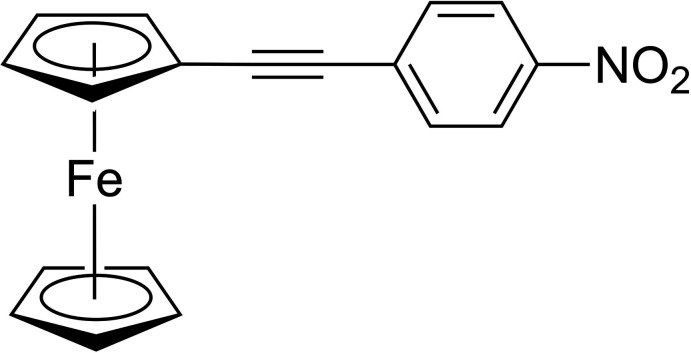


## Structural commentary   

**Figure 1:**
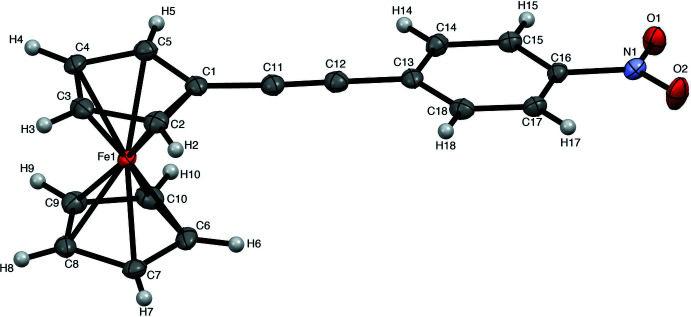
Mol­ecular structure of the title compound, including atom labelling. Displacement ellipsoids are drawn at the 50% probability level.

Fig. 1 ▸ (*Mercury*; Macrae *et al.*, 2020 ▸) shows the mol­ecular structure of the title compound, which crystallizes in space group *P*2_1_/*n*. The substituted ferrocene (Fc) system is linked to a *p*-nitro­benzene moiety by an acetyl­enic bridge between C11 and C12 with a bond distance of 1.202 (2) Å, which is comparable to those in similar complexes, *e.g.* 1.202 (2) Å (Misra *et al.* 2014 ▸), 1.197 (3) Å (Fu *et al.*, 2008 ▸), and 1.193 (2) Å (Zora *et al.* 2006 ▸). The unit cell is comprised of four mol­ecules with one mol­ecule present per asymmetric unit. The substituted Cp and phenyl rings are almost parallel to each other, subtending a dihedral angle of 6.19 (10)°, in contrast to (phenyl-ethyn­yl)ferrocene (Zora *et al.*, 2006 ▸), which has no substituent in the *para* position and exhibits a nearly perpendicular dihedral angle of 89.06 (3)°. The distances of the Fe1 atom from the centroids of the substituted and unsubstituted Cp rings are 1.6461 (8) and 1.6584 (8) Å, respectively. The *Cg*1—Fe1—*Cg*2 angle is 179.27°, where *Cg*1 and *Cg*2 are the centroids of substituted and unsubstituted Cp rings, respectively. The Cp rings in the ferrocene system are thus almost parallel, since the angle between the Cp ring planes is 1.03 (13)°. In addition, the Cp rings display a nearly eclipsed conformation with a slight deviation, as demonstrated by the average C—*Cg*1—*Cg*2—C torsion angle of 12.26°. The C—C bond distances in the Cp rings range from 1.417 (2) to 1.436 (2) Å, while the Fe—C bond lengths range between 2.038 (2) and 2.055 (2) Å.

## Supra­molecular features   

**Figure 2:**
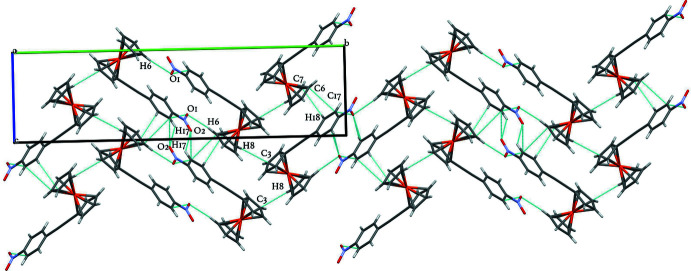
Crystal packing of the title compound along the *a* axis with short-contact inter­actions shown as dashed lines.

The title compound exhibits π–π stacking inter­actions between one of the Cp rings from the Fc moiety and the *p*-nitro­phenyl substituent, allowing the formation of a zigzag structure; atom pairs involved relate C6(Cp) and C7(Cp) to C17(*p*-nitro­phen­yl) and C18(*p*-nitro­phen­yl) of a neighboring mol­ecule, with short contacts of 3.340 (2) and 3.397 (2) Å, respectively. This inter­action can be described as pairs of mol­ecules being inter­rupted by two C3(Cp)⋯H8—C8(Cp) inter­actions from a different inter­connected pair of perpendicularly oriented Fc moieties with short contact distances of 2.83 Å each. Short contacts from neighboring mol­ecules establishing a distinctive inter­connected pair between a corner of the Cp ring and one of the oxygen atoms from the *p*-nitro­phenyl substituent yield a closed arrangement of atoms. Short contacts involve H6—C6(Cp)⋯O1(*p*-nitro­phen­yl) at a distance of 3.461 (2) Å. Another inter­connection is found between adjacent *p*-nitro­phenyl groups, yielding a ring arrangement involving pairs from H17—C17(*p*-nitro­phen­yl)⋯O2(*p*-nitro­phen­yl) with a distance of 2.727 (2) Å and pairs from O1(*p*-nitro­phen­yl)⋯H15—C15(*p*-nitro­phen­yl) with a distance of 2.716 (2) Å. In addition, a chain is formed by short contacts from the C17—H17(*p*-nitro­phen­yl)⋯O1(*p*-nitro­phen­yl) inter­action belonging to the *p*-nitro­phenyl substituent with a distance of 3.203 (19) Å. Numerical details of the hydrogen-bonding inter­actions are given in Table 1 ▸ and the packing is shown in Fig. 2 ▸.

## Hirshfeld Surface Analysis   

**Figure 3:**
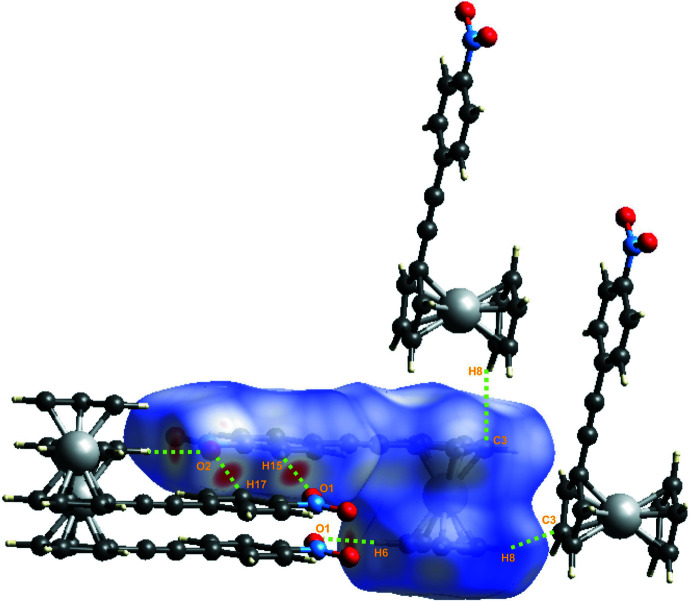
A view of the Hirshfeld surface of the title compound mapped over *d*_norm_ with the four main inter­molecular contacts in the crystal lattice.

**Figure 4:**
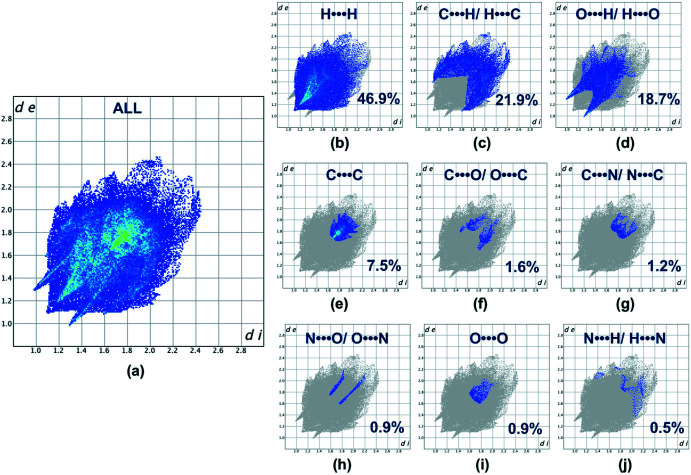
Full (*a*) and individual (*b*)–(*j*) two-dimensional fingerprint plots showing the nine inter­molecular contacts present in the crystal structure.

*CrystalExplorer17* (Turner *et al.*, 2017 ▸) was used to generate the Hirshfeld surface (Spackman & Jayatilaka, 2009 ▸) for the title compound mapped over *d*_norm_ and the associated two-dimensional fingerprint plots (McKinnon *et al.*, 2007 ▸). Fig. 3 ▸ shows the mol­ecules involved in the four closest contacts. Red spots on the Hirshfeld surface mapped over *d*_norm_ in the color range −0.2315 to 1.1417 arbitrary units confirm the previously mentioned main inter­molecular contacts. The fingerprint plots are given for all contacts (Fig. 4 ▸ *a*) and those decomposed into nine individual inter­actions: H⋯H (46.9%; Fig. 4 ▸ *b*), C⋯H/ H⋯C (21.9%; Fig. 4 ▸ *c*), O⋯H/ H⋯O (18.7%; Fig. 4 ▸ *d*), C⋯C (7.5%; Fig. 4 ▸ *e*), C⋯O/O⋯C (1.6%; Fig. 4 ▸ *f*), C⋯N/N⋯C (1.2%; Fig. 4 ▸ *g*), N⋯O/O⋯N (0.9%; Fig. 4 ▸ *h*), O⋯O (0.9%; Fig. 4 ▸ *i*) and N⋯H/H⋯N (0.5%; Fig. 4 ▸ *j*). The Hirshfeld surface analysis for the title compound indicates that the most significant contributions arise from H⋯H and C⋯H contacts (González *et al.*, 2020 ▸; McKinnon *et al.*, 2004 ▸, 2007 ▸; Spackman & McKinnon, 2002 ▸).

## Database survey   

A search of the Cambridge Structural Database (Version 5.41, updated November 2019; Groom *et al.*, 2016 ▸) revealed 142 related compounds with the 1-ferrocenyl-2-phenyl­ethyne backbone. Of those structures, 41 contain substituents in the *para* position of the phenyl ring, as in the title compound. One of the reasons for such a high number of reported structures for methynylferrocene and its derived compounds is attributed to their substantial inter­est as chromophores, mainly because of their electronic communication capacity through the alkyne linkage to the Fe center. When comparing the effect of the substituent on the mol­ecular structure, one of the main features is the dihedral angle that is formed between the substituted Cp ring of the ferrocene group and the phenyl moiety. The orientation can range from almost parallel (1.01°: YOHSIY; Bobula *et al.*, 2008 ▸) to completely perpendicular (90.00°: YOHSUK01; Dai *et al.*, 2013 ▸). Table 2 ▸ gives the dihedral angles for previously reported compounds; our compound having the second lowest dihedral angle and a nearly parallel conformation. Exchanging the hydrogen atoms in the methyl group for fluorine atoms shifts the dihedral angle from 1.01° to 90.00° in the case of methyl and tri­fluoro­methyl substituents, respectively.

## Synthesis and crystallization   

The title compound was prepared by adding ethynylferrocene (1.0 mmol), PdCl_2_(PPh_3_)_2_ (0.01 mmol), Et_3_N (2 mmol) and 4-bromo-1-nitro­benzene (1.0 mmol) to a 25 mL round-bottom flask, followed by the addition of DMF (1.0 mL) by syringe. The reaction was stirred for 1 h at 353 K. The reaction was stopped and crashed out with 20 mL of cold distilled water, then the solid was vacuum filtrated, and chromatographed [silica (hepta­ne–ethyl acetate/7:3)] to afford the pure compound, 70% yield. Dark-red crystals suitable for X-ray diffraction were obtained by the slow evaporation of CDCl_3_ solution of the title compound at room temperature. NMR analyses were performed on a Bruker AV-700 spectrometer by using CDCl_3_ 99.9% pure as a solvent and Me_4_Si as external standard.^1^H NMR (δ in ppm, CDCl_3_): 4.26 (*s*, 5H), 4.32 (*s*, 2H), 4.55 (*s*, 2H), 7.59 (*d*, *J* = 8.6Hz, 2H), 8.18 (*d*, *J* = 8.6Hz, 2H). ^13^C NMR (δ in ppm, CDCl_3_): 63.6, 69.6, 70.1, 71.8, 84.5, 95.2, 123.6, 131.1, 131.8, 146.4. IR (ν_max_, cm^−1^): 2200 (C≡C). Electrochemistry: (CV_200 mv_: *E* ^o^ = 613 mV; Δ*E* = 90 mV).

## Refinement   

Crystal data, data collection and structure refinement details are summarized in Table 3 ▸. H atoms were included in geometrically calculated positions, C—H = 0.93 Å, and refined as riding on their parent C atom with *U*_iso_(H) = 1.2*U*_eq_(C).

## Supplementary Material

Crystal structure: contains datablock(s) I. DOI: 10.1107/S2056989020010336/dj2003sup1.cif

Structure factors: contains datablock(s) I. DOI: 10.1107/S2056989020010336/dj2003Isup2.hkl

CCDC reference: 1991635

Additional supporting information:  crystallographic information; 3D view; checkCIF report

## Figures and Tables

**Table 1:** Hydrogen-bond geometry (Å, °)

*D*—H⋯*A*	*D*—H	H⋯*A*	*D*⋯*A*	*D*—H⋯*A*
C6—H6⋯O1^i^	0.93	2.55	3.461 (2)	168
C15—H15⋯O1^ii^	0.93	2.41	3.1909 (19)	141
C17—H17⋯O2^iii^	0.93	2.49	3.2187 (19)	135

Symmetry codes: (i) 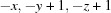; (ii) 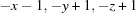; (iii) 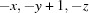.

**Table 2:** The effect of the substituent on the dihedral angle (°) between the substituted Cp ring and the phenyl ring in compounds containing a 1-ferrocenyl-2-phenyl­ethyne backbone and a *para*-substituted phenyl ring

Substituent	Dihedral angle	Refcode
Methyl (CH_3_)	1.01 (9)	YOHSIY (Bobula *et al.*, 2008 ▸)
Nitro (NO_2_)	6.61 (9)	This work
Amino (NH_2_)	8.05 (9)	YONFEN (Siemeling *et al.*, 2008 ▸)
Ethynyl (C≡CH)	8.61 (9)	RARNED (Lin *et al.*, 1996 ▸)
Iodo (I)	37.25 (9)	GIZTOA (Misra *et al.*, 2014 ▸)
Cyano (C≡N)	69.58 (9)	MIJLAS01 (Bobula *et al.*, 2008 ▸)
Hydrogen (H)	89.06 (9)	KELTIF (Zora *et al.*, 2006 ▸)
Tri­fluoro­methyl (CF_3_)	90.00 (9)	YOKSUK01 (Dai *et al.*, 2013 ▸)

**Table 3:** Experimental details

Crystal data	
Chemical formula	[Fe(C_5_H_5_)_2_(C_8_NO_2_)]
*M*_r_	331.14
Crystal system, space group	Monoclinic, *P*2_1_/*n*
Temperature (K)	100
*a*, *b*, *c* (Å)	5.9573 (1), 29.3810 (3), 8.0664 (1)
β (°)	100.202 (1)
*V* (Å^3^)	1389.55 (3)
*Z*	4
Radiation type	Cu *K*α
μ (mm^−1^)	8.75
Crystal size (mm)	0.20 × 0.07 × 0.04

Data collection	
Diffractometer	Rigaku SuperNova, Single source at offset/far, HyPix3000
Absorption correction	Multi-scan (*CrysAlis PRO*; Rigaku OD, 2018 ▸)
*T*_min_, *T*_max_	0.642, 1.000
No. of measured, independent and observed [*I* > 2σ(*I*)] reflections	21951, 2567, 2410
*R*_int_	0.036
(sin θ/λ)_max_ (Å^−1^)	0.605

Refinement	
*R*[*F* ^2^ > 2σ(*F* ^2^)], *wR*(*F* ^2^), *S*	0.024, 0.061, 1.07
No. of reflections	2567
No. of parameters	200
H-atom treatment	H-atom parameters constrained
Δρ_max_, Δρ_min_ (e Å^−3^)	0.23, −0.37

Computer programs: *CrysAlis PRO* (Rigaku OD, 2018 ▸), *SHELXT* (Sheldrick, 2015*a* ▸), *SHELXL* (Sheldrick, 2015*b* ▸) and *OLEX2* (Dolomanov *et al.*, 2009 ▸).

## supplementary crystallographic information

### Crystal data

**Table:**

[Fe(C_5_H_5_)_2_(C_8_NO_2_)]	*F*(000) = 680
*M**_r_* = 331.14	*D*_x_ = 1.583 Mg m^−^^3^
Monoclinic, *P*2_1_/*n*	Cu *K*α radiation, λ = 1.54184 Å
*a* = 5.9573 (1) Å	Cell parameters from 12369 reflections
*b* = 29.3810 (3) Å	θ = 3.0–68.8°
*c* = 8.0664 (1) Å	µ = 8.75 mm^−^^1^
β = 100.202 (1)°	*T* = 100 K
*V* = 1389.55 (3) Å^3^	Block, dark red
*Z* = 4	0.20 × 0.07 × 0.04 mm

### Data collection

**Table:**

Rigaku SuperNova, Single source at offset/far, HyPix3000 diffractometer	2567 independent reflections
Radiation source: micro-focus sealed X-ray tube, SuperNova (Cu) X-ray Source	2410 reflections with *I* > 2σ(*I*)
Mirror monochromator	*R*_int_ = 0.036
ω scans	θ_max_ = 68.9°, θ_min_ = 3.0°
Absorption correction: multi-scan (CrysAlisPro; Rigaku OD, 2018)	*h* = −7→7
*T*_min_ = 0.642, *T*_max_ = 1.000	*k* = −35→35
21951 measured reflections	*l* = −9→9

### Refinement

**Table:**

Refinement on *F*^2^	Hydrogen site location: inferred from neighbouring sites
Least-squares matrix: full	H-atom parameters constrained
*R*[*F*^2^ > 2σ(*F*^2^)] = 0.024	*w* = 1/[σ^2^(*F*_o_^2^) + (0.0305*P*)^2^ + 0.6401*P*] where *P* = (*F*_o_^2^ + 2*F*_c_^2^)/3
*wR*(*F*^2^) = 0.061	(Δ/σ)_max_ = 0.003
*S* = 1.07	Δρ_max_ = 0.23 e Å^−^^3^
2567 reflections	Δρ_min_ = −0.37 e Å^−^^3^
200 parameters	Extinction correction: SHELXL-2018/3 (Sheldrick 2015b), Fc^*^=kFc[1+0.001xFc^2^λ^3^/sin(2θ)]^-1/4^
0 restraints	Extinction coefficient: 0.00060 (13)
Primary atom site location: dual	

### Special details

**Table:**

Geometry. All esds (except the esd in the dihedral angle between two l.s. planes) are estimated using the full covariance matrix. The cell esds are taken into account individually in the estimation of esds in distances, angles and torsion angles; correlations between esds in cell parameters are only used when they are defined by crystal symmetry. An approximate (isotropic) treatment of cell esds is used for estimating esds involving l.s. planes.

### Fractional atomic coordinates and isotropic or equivalent isotropic displacement parameters (Å^2^)

**Table:**

	*x*	*y*	*z*	*U*_iso_*/*U*_eq_	
Fe1	0.76257 (4)	0.67811 (2)	0.90888 (3)	0.01397 (9)	
O1	−0.49667 (19)	0.47779 (4)	0.27187 (15)	0.0226 (3)	
O2	−0.2775 (2)	0.46760 (4)	0.08806 (15)	0.0271 (3)	
N1	−0.3245 (2)	0.48668 (4)	0.21343 (17)	0.0167 (3)	
C1	0.5321 (3)	0.68695 (5)	0.6915 (2)	0.0173 (3)	
C2	0.7551 (3)	0.70112 (5)	0.6686 (2)	0.0192 (3)	
H2	0.837507	0.689297	0.590834	0.023*	
C3	0.8274 (3)	0.73649 (5)	0.7863 (2)	0.0208 (3)	
H3	0.965574	0.751951	0.798205	0.025*	
C4	0.6531 (3)	0.74426 (5)	0.8827 (2)	0.0205 (3)	
H4	0.657556	0.765623	0.968280	0.025*	
C5	0.4711 (3)	0.71379 (5)	0.8256 (2)	0.0198 (3)	
H5	0.335922	0.711567	0.867718	0.024*	
C6	0.7951 (3)	0.60950 (5)	0.9567 (2)	0.0221 (4)	
H6	0.732434	0.586180	0.885223	0.026*	
C7	1.0133 (3)	0.62957 (5)	0.9617 (2)	0.0193 (3)	
H7	1.118391	0.621784	0.893815	0.023*	
C8	1.0425 (3)	0.66366 (5)	1.0887 (2)	0.0192 (3)	
H8	1.170282	0.682060	1.118684	0.023*	
C9	0.8431 (3)	0.66474 (6)	1.1617 (2)	0.0212 (4)	
H9	0.817060	0.683939	1.247992	0.025*	
C10	0.6897 (3)	0.63126 (6)	1.0798 (2)	0.0233 (4)	
H10	0.545564	0.624788	1.103002	0.028*	
C11	0.3932 (3)	0.65207 (5)	0.6029 (2)	0.0189 (3)	
C12	0.2680 (3)	0.62353 (6)	0.5308 (2)	0.0195 (3)	
C13	0.1182 (3)	0.58890 (5)	0.4500 (2)	0.0170 (3)	
C14	−0.0833 (3)	0.57845 (5)	0.5091 (2)	0.0182 (3)	
H14	−0.119391	0.594223	0.600861	0.022*	
C15	−0.2291 (3)	0.54491 (5)	0.43247 (19)	0.0167 (3)	
H15	−0.363101	0.537993	0.471276	0.020*	
C16	−0.1700 (3)	0.52188 (5)	0.2961 (2)	0.0153 (3)	
C17	0.0281 (3)	0.53113 (5)	0.2348 (2)	0.0170 (3)	
H17	0.063821	0.515003	0.143683	0.020*	
C18	0.1713 (3)	0.56480 (5)	0.3118 (2)	0.0182 (3)	
H18	0.304440	0.571598	0.271639	0.022*	

### Atomic displacement parameters (Å^2^)

**Table:**

	*U*^11^	*U*^22^	*U*^33^	*U*^12^	*U*^13^	*U*^23^
Fe1	0.01496 (14)	0.01010 (14)	0.01622 (15)	−0.00021 (9)	0.00100 (10)	0.00119 (9)
O1	0.0200 (6)	0.0236 (6)	0.0264 (6)	−0.0064 (5)	0.0101 (5)	−0.0040 (5)
O2	0.0305 (7)	0.0265 (7)	0.0274 (7)	−0.0059 (5)	0.0137 (6)	−0.0127 (5)
N1	0.0188 (7)	0.0139 (6)	0.0179 (7)	0.0005 (5)	0.0049 (5)	0.0000 (5)
C1	0.0184 (8)	0.0138 (7)	0.0183 (8)	0.0005 (6)	−0.0004 (7)	0.0045 (6)
C2	0.0213 (8)	0.0158 (8)	0.0207 (8)	−0.0001 (6)	0.0039 (7)	0.0054 (6)
C3	0.0187 (8)	0.0142 (8)	0.0278 (9)	−0.0020 (6)	−0.0008 (7)	0.0071 (7)
C4	0.0233 (9)	0.0117 (7)	0.0243 (9)	0.0027 (6)	−0.0015 (7)	0.0004 (6)
C5	0.0180 (8)	0.0163 (8)	0.0244 (9)	0.0023 (6)	0.0020 (7)	0.0025 (6)
C6	0.0282 (9)	0.0111 (8)	0.0236 (9)	−0.0010 (6)	−0.0047 (7)	0.0037 (6)
C7	0.0216 (8)	0.0149 (8)	0.0205 (8)	0.0050 (6)	0.0011 (7)	0.0021 (6)
C8	0.0201 (8)	0.0151 (8)	0.0201 (8)	−0.0003 (6)	−0.0024 (7)	0.0021 (6)
C9	0.0274 (9)	0.0200 (8)	0.0161 (8)	0.0035 (7)	0.0030 (7)	0.0022 (6)
C10	0.0203 (8)	0.0229 (9)	0.0262 (9)	−0.0016 (7)	0.0026 (7)	0.0106 (7)
C11	0.0200 (8)	0.0165 (8)	0.0196 (8)	0.0012 (6)	0.0014 (7)	0.0032 (6)
C12	0.0203 (8)	0.0177 (8)	0.0200 (8)	0.0017 (7)	0.0026 (7)	0.0037 (7)
C13	0.0190 (8)	0.0131 (7)	0.0176 (8)	0.0007 (6)	−0.0005 (6)	0.0041 (6)
C14	0.0225 (8)	0.0166 (8)	0.0155 (8)	0.0018 (6)	0.0032 (6)	−0.0001 (6)
C15	0.0176 (8)	0.0164 (8)	0.0165 (8)	0.0010 (6)	0.0045 (6)	0.0022 (6)
C16	0.0175 (8)	0.0123 (7)	0.0159 (8)	−0.0002 (6)	0.0019 (6)	0.0011 (6)
C17	0.0183 (8)	0.0162 (8)	0.0169 (8)	0.0023 (6)	0.0048 (6)	0.0006 (6)
C18	0.0172 (8)	0.0177 (8)	0.0200 (8)	0.0007 (6)	0.0038 (6)	0.0045 (6)

### Geometric parameters (Å, º)

**Table:**

Fe1—C1	2.0431 (17)	C6—H6	0.9300
Fe1—C2	2.0454 (16)	C6—C7	1.422 (2)
Fe1—C3	2.0505 (16)	C6—C10	1.418 (3)
Fe1—C4	2.0491 (16)	C7—H7	0.9300
Fe1—C5	2.0375 (16)	C7—C8	1.421 (2)
Fe1—C6	2.0552 (16)	C8—H8	0.9300
Fe1—C7	2.0546 (16)	C8—C9	1.417 (2)
Fe1—C8	2.0515 (17)	C9—H9	0.9300
Fe1—C9	2.0489 (17)	C9—C10	1.423 (3)
Fe1—C10	2.0488 (16)	C10—H10	0.9300
O1—N1	1.2301 (17)	C11—C12	1.202 (2)
O2—N1	1.2312 (17)	C12—C13	1.432 (2)
N1—C16	1.464 (2)	C13—C14	1.402 (2)
C1—C2	1.435 (2)	C13—C18	1.403 (2)
C1—C5	1.437 (2)	C14—H14	0.9300
C1—C11	1.427 (2)	C14—C15	1.385 (2)
C2—H2	0.9300	C15—H15	0.9300
C2—C3	1.422 (2)	C15—C16	1.389 (2)
C3—H3	0.9300	C16—C17	1.385 (2)
C3—C4	1.422 (2)	C17—H17	0.9300
C4—H4	0.9300	C17—C18	1.380 (2)
C4—C5	1.418 (2)	C18—H18	0.9300
C5—H5	0.9300		
			
C1—Fe1—C2	41.09 (6)	C4—C3—C2	108.54 (14)
C1—Fe1—C3	68.60 (6)	C4—C3—H3	125.7
C1—Fe1—C4	68.76 (6)	Fe1—C4—H4	126.6
C1—Fe1—C6	108.20 (7)	C3—C4—Fe1	69.76 (9)
C1—Fe1—C7	128.28 (7)	C3—C4—H4	125.9
C1—Fe1—C8	166.33 (7)	C5—C4—Fe1	69.25 (9)
C1—Fe1—C9	151.89 (7)	C5—C4—C3	108.16 (15)
C1—Fe1—C10	118.21 (7)	C5—C4—H4	125.9
C2—Fe1—C3	40.62 (7)	Fe1—C5—H5	125.9
C2—Fe1—C4	68.63 (7)	C1—C5—Fe1	69.60 (9)
C2—Fe1—C6	119.18 (7)	C1—C5—H5	126.0
C2—Fe1—C7	108.59 (7)	C4—C5—Fe1	70.13 (9)
C2—Fe1—C8	128.09 (7)	C4—C5—C1	108.06 (15)
C2—Fe1—C9	165.58 (7)	C4—C5—H5	126.0
C2—Fe1—C10	152.64 (7)	Fe1—C6—H6	126.3
C3—Fe1—C6	153.07 (7)	C7—C6—Fe1	69.74 (9)
C3—Fe1—C7	119.24 (7)	C7—C6—H6	126.0
C3—Fe1—C8	108.37 (7)	C10—C6—Fe1	69.55 (9)
C4—Fe1—C3	40.58 (7)	C10—C6—H6	126.0
C4—Fe1—C6	165.33 (7)	C10—C6—C7	108.03 (15)
C4—Fe1—C7	152.40 (7)	Fe1—C7—H7	126.1
C4—Fe1—C8	118.25 (7)	C6—C7—Fe1	69.78 (9)
C5—Fe1—C1	41.23 (7)	C6—C7—H7	126.1
C5—Fe1—C2	69.11 (7)	C8—C7—Fe1	69.63 (9)
C5—Fe1—C3	68.47 (7)	C8—C7—C6	107.88 (15)
C5—Fe1—C4	40.62 (7)	C8—C7—H7	126.1
C5—Fe1—C6	127.80 (7)	Fe1—C8—H8	126.1
C5—Fe1—C7	166.23 (7)	C7—C8—Fe1	69.87 (9)
C5—Fe1—C8	151.41 (7)	C7—C8—H8	125.9
C5—Fe1—C9	117.54 (7)	C9—C8—Fe1	69.69 (9)
C5—Fe1—C10	107.31 (7)	C9—C8—C7	108.12 (15)
C7—Fe1—C6	40.48 (7)	C9—C8—H8	125.9
C8—Fe1—C6	68.07 (7)	Fe1—C9—H9	126.0
C8—Fe1—C7	40.50 (6)	C8—C9—Fe1	69.89 (9)
C9—Fe1—C3	127.56 (7)	C8—C9—H9	126.0
C9—Fe1—C4	107.38 (7)	C8—C9—C10	107.97 (15)
C9—Fe1—C6	68.12 (7)	C10—C9—Fe1	69.67 (9)
C9—Fe1—C7	68.10 (7)	C10—C9—H9	126.0
C9—Fe1—C8	40.42 (7)	Fe1—C10—H10	125.9
C10—Fe1—C3	165.28 (7)	C6—C10—Fe1	70.03 (9)
C10—Fe1—C4	127.24 (7)	C6—C10—C9	108.00 (15)
C10—Fe1—C6	40.42 (7)	C6—C10—H10	126.0
C10—Fe1—C7	68.11 (7)	C9—C10—Fe1	69.68 (9)
C10—Fe1—C8	68.14 (7)	C9—C10—H10	126.0
C10—Fe1—C9	40.65 (7)	C12—C11—C1	177.07 (18)
O1—N1—O2	123.00 (13)	C11—C12—C13	178.15 (18)
O1—N1—C16	118.36 (13)	C14—C13—C12	120.19 (15)
O2—N1—C16	118.63 (13)	C14—C13—C18	119.13 (15)
C2—C1—Fe1	69.54 (9)	C18—C13—C12	120.67 (15)
C2—C1—C5	107.51 (14)	C13—C14—H14	119.6
C5—C1—Fe1	69.18 (9)	C15—C14—C13	120.74 (15)
C11—C1—Fe1	125.49 (11)	C15—C14—H14	119.6
C11—C1—C2	127.74 (15)	C14—C15—H15	120.9
C11—C1—C5	124.74 (15)	C14—C15—C16	118.24 (14)
Fe1—C2—H2	126.2	C16—C15—H15	120.9
C1—C2—Fe1	69.37 (9)	C15—C16—N1	118.51 (14)
C1—C2—H2	126.1	C17—C16—N1	118.86 (14)
C3—C2—Fe1	69.88 (9)	C17—C16—C15	122.62 (15)
C3—C2—C1	107.72 (14)	C16—C17—H17	120.7
C3—C2—H2	126.1	C18—C17—C16	118.53 (15)
Fe1—C3—H3	126.7	C18—C17—H17	120.7
C2—C3—Fe1	69.50 (9)	C13—C18—H18	119.6
C2—C3—H3	125.7	C17—C18—C13	120.73 (15)
C4—C3—Fe1	69.66 (9)	C17—C18—H18	119.6
			
Fe1—C1—C2—C3	−59.59 (11)	C5—C1—C2—C3	−0.63 (18)
Fe1—C1—C5—C4	59.82 (11)	C6—C7—C8—Fe1	−59.53 (11)
Fe1—C2—C3—C4	−58.88 (11)	C6—C7—C8—C9	−0.12 (18)
Fe1—C3—C4—C5	−58.78 (11)	C7—C6—C10—Fe1	59.33 (11)
Fe1—C4—C5—C1	−59.48 (11)	C7—C6—C10—C9	−0.24 (18)
Fe1—C6—C7—C8	59.43 (11)	C7—C8—C9—Fe1	−59.51 (11)
Fe1—C6—C10—C9	−59.57 (12)	C7—C8—C9—C10	−0.02 (18)
Fe1—C7—C8—C9	59.40 (11)	C8—C9—C10—Fe1	−59.63 (11)
Fe1—C8—C9—C10	59.49 (11)	C8—C9—C10—C6	0.16 (19)
Fe1—C9—C10—C6	59.79 (11)	C10—C6—C7—Fe1	−59.21 (11)
O1—N1—C16—C15	−2.5 (2)	C10—C6—C7—C8	0.22 (18)
O1—N1—C16—C17	177.91 (14)	C11—C1—C2—Fe1	−119.65 (17)
O2—N1—C16—C15	176.88 (14)	C11—C1—C2—C3	−179.24 (15)
O2—N1—C16—C17	−2.7 (2)	C11—C1—C5—Fe1	119.48 (16)
N1—C16—C17—C18	179.07 (14)	C11—C1—C5—C4	179.29 (15)
C1—C2—C3—Fe1	59.27 (11)	C12—C13—C14—C15	−179.76 (15)
C1—C2—C3—C4	0.39 (18)	C12—C13—C18—C17	179.41 (15)
C2—C1—C5—Fe1	−59.19 (11)	C13—C14—C15—C16	0.2 (2)
C2—C1—C5—C4	0.62 (18)	C14—C13—C18—C17	−0.2 (2)
C2—C3—C4—Fe1	58.78 (11)	C14—C15—C16—N1	−179.41 (14)
C2—C3—C4—C5	−0.01 (18)	C14—C15—C16—C17	0.1 (2)
C3—C4—C5—Fe1	59.10 (11)	C15—C16—C17—C18	−0.4 (2)
C3—C4—C5—C1	−0.38 (18)	C16—C17—C18—C13	0.5 (2)
C5—C1—C2—Fe1	58.96 (11)	C18—C13—C14—C15	−0.2 (2)

### Hydrogen-bond geometry (Å, º)

**Table:**

*D*—H···*A*	*D*—H	H···*A*	*D*···*A*	*D*—H···*A*
C6—H6···O1^i^	0.93	2.55	3.461 (2)	168
C15—H15···O1^ii^	0.93	2.41	3.1909 (19)	141
C17—H17···O2^iii^	0.93	2.49	3.2187 (19)	135

Symmetry codes: (i) −*x*, −*y*+1, −*z*+1; (ii) −*x*−1, −*y*+1, −*z*+1; (iii) −*x*, −*y*+1, −*z*.
